# A Cosmetic Approach to Cheek and Nasal Hemangioma Utilizing Open Rhinoplasty in a 10-Year-Old Child: A Case Report

**DOI:** 10.7759/cureus.34185

**Published:** 2023-01-25

**Authors:** Mohamed A Mrad, Felwa A AlMarshad, Abdullah M AlZahrani, Dana A Obeid, Tuqa A Alsinan, Ahmad S AlOtaibi

**Affiliations:** 1 Plastic and Reconstructive Surgery Section, Department of Surgery, King Faisal Specialist Hospital and Research Centre, Riyadh, SAU; 2 Otolaryngology - Head and Neck Surgery, King Saud Bin Abdulaziz University for Health Sciences College of Medicine, Riyadh, SAU; 3 Plastic and Reconstructive Surgery, King Saud Medical City, Riyadh, SAU

**Keywords:** pediatric plastic surgery, open rhinoplasty, hemangioma, facial cosmetic surgery, congenital malformation

## Abstract

Infantile hemangiomas are characterized as benign tumors of vascular tissue that arise from rapid endothelial cell proliferation followed by gradual involution, affecting 4% to 5% in infants and 2.6% to 9.9% in older children. Most of them resolve by the age of three years, negating the need for surgical intervention. However, intervention should be considered especially in cases with a high risk of recurrence. A female patient, aged 10 years, was referred to plastic surgery by her dermatologist due to the presence of a vascular mass in her face located at the junction between the nose and right cheek that had been present since infancy. The patient was diagnosed with infantile hemangioma based on MRI imaging of the face showing a benign vascular lesion measuring 9 x 12 mm. After the failure of multiple sclerotherapy sessions and informed discussion with the respective family, the patient underwent open rhinoplasty for surgical excision with no facial scarring other than the transcellular scar. This study presents a rare case of utilizing the open rhinoplasty technique in a relapsing facial hemangioma of a 10-year-old child. Results show a positive aesthetic outcome by minimizing facial scars. Considering the limited reported use of this technique, more clinical studies, especially comparing long-term effects across different age populations, are recommended to validate the efficiency and effectiveness of this technique.

## Introduction

Infantile hemangioma (IH) is the most common pediatric vascular tumor. It is estimated to affect 4% of infants though the reported incidence ranges from 2-10% [1-4]. As infants are usually discharged before IH becomes apparent, registry data may be incomplete.

IH usually occurs during the first few weeks of life and follows a typical natural path of proliferation and involution. Their clinical appearance depends on their depth and distribution. Its pathogenesis is not well-known but is thought to represent an abnormal response of pluripotent stem cells to stimuli such as hypoxia and the renin-angiotensin system.

Classification comprises superficial, mixed, and deep IH as well as IH with minimal or arrested growth.

Large facial or lumbar IH permits investigation for the PHACE (Posterior fossa anomalies, Hemangioma, Arterial anomalies, Cardiac anomalies, Eye anomalies, and Sternal defects) and LUMBAR (Lower body hemangioma, Urogenital abnormalities/ulceration, Myelopathy, Bony deformities, Anorectal malformations/arterial anomalies, and Rectal anomalies) syndromes, respectively. The complications of IH include ulceration, obstruction or functional impairment, hypothyroidism, and cosmetic sequelae.

In this case report, we present a child with facial hemangioma treated by open rhinoplasty.

## Case presentation

**Figure 1:**
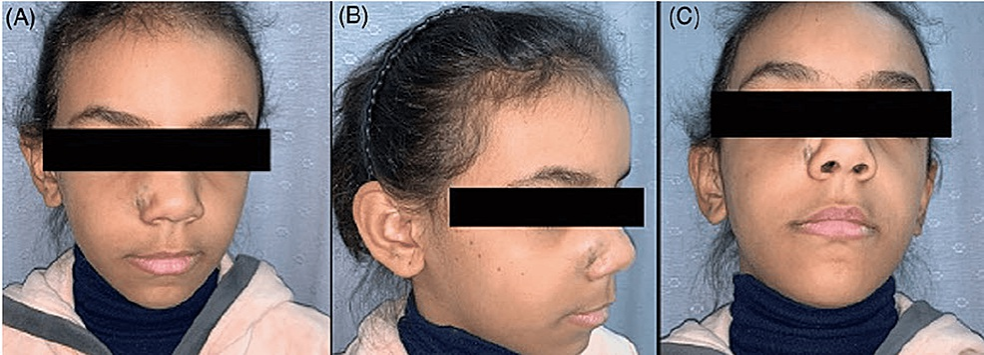
Preoperative images of the patient

**Figure 2:**
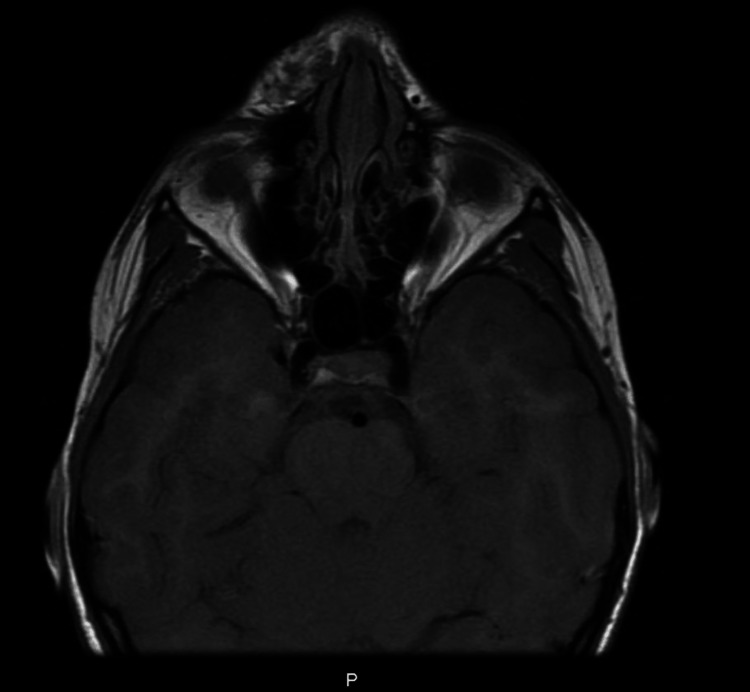
MRI scan of the facial soft tissue with contrast (white arrow) showing vascular lesion centered in the right nasal ala and nasolabial fold

**Figure 3:**
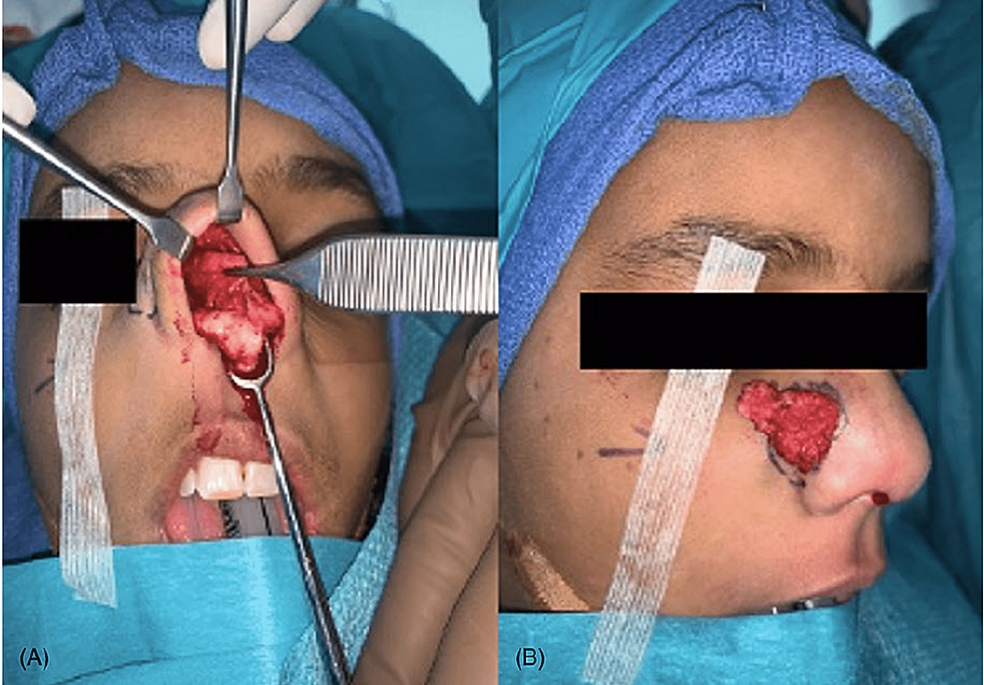
Intraoperative appearance of the vascular mass with scar tissues around it

**Figure 4:**
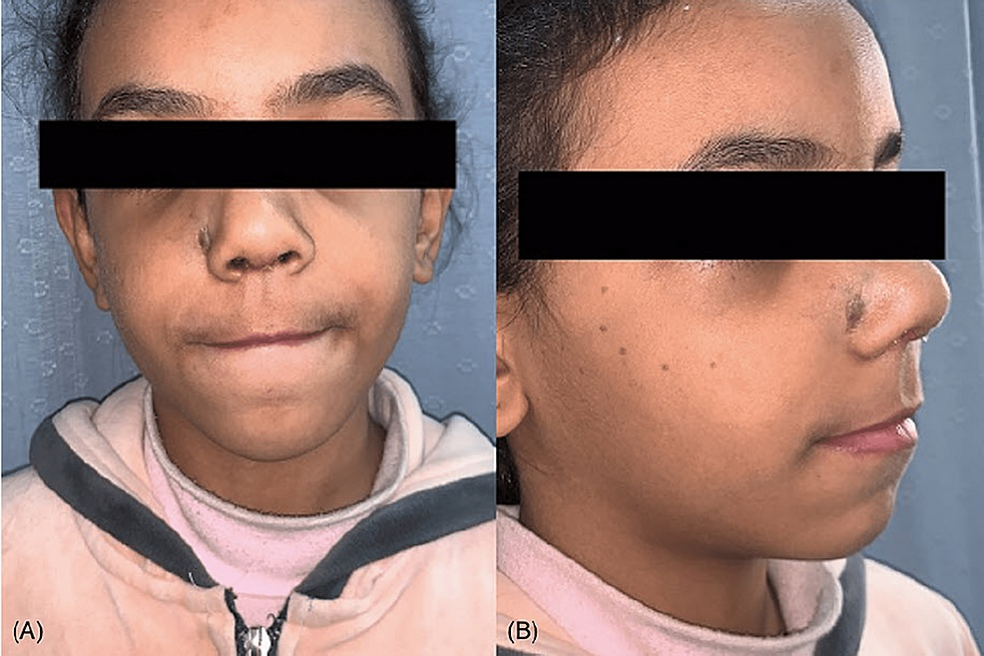
Facial appearance showing minimal scarring after two weeks of follow-up

Our patient was a 10-year-old Saudi female who presented to the senior author (MAM) and was diagnosed with congenital nasal hemangioma. She is the third child of healthy parents. The pregnancy was uncomplicated, and the family history was negative for any congenital anomalies. Prior to her presentation, the patient was treated previously by oral propranolol by her dermatologist. On clinical examination, the patient had a vascular mass on the face located at the junction between the nose and right cheek (Figures 1A-1C). Radiological investigations by means of magnetic resonance imaging (MRI) of the face confirmed the presence of a benign vascular lesion measuring 9 x 12 mm centered posterior to the right nasal ala, upper one-third of the nasolabial fold, and right side of the upper lip with no invasion to the nasal septum (Figure 2). The surgical option was offered to the family, and they accepted the surgical intervention and its complications. The patient was prepared for surgical excision under general anesthesia. A stair-step incision at the waist of the nasal columella was performed. This was followed by bilateral marginal incisions. The nasal skin flap was raised over the lower lateral cartilages and then extended laterally to the alar facial groove until good exposure of the mass was achieved. Operative findings were found similar to the radiological ones. The mass was dissected from surrounding tissues down to the piriform aperture and anterior wall of the maxilla (Figures 3A, 3B). The mass excised completely, maintaining all surrounding normal tissue. Hemostasis was achieved and then closed with 6 - 0 and 5 - 0 absorbable sutures for the trans columellar and marginal incisions, respectively. Nasal taping and a small nasal splint were applied. The patient tolerated the procedure very well with no early or late complications. After two weeks of follow-up, the patient and her family reported good aesthetics and satisfactory outcomes (Figures 4A, 4B).

## Discussion

The typical presentation of infantile hemangioma is a solid vascularized tumor with cutaneous lesions that may present as red patches on any part of the body and usually appear after two weeks of age [5]. The diagnosis of infantile hemangioma is based on clinical presentation and rarely needs imaging. The tumor rapidly grows until the patient is nine to 12 months old, termed the proliferating phase, and usually regresses after 3.5 years of age during the involuting phase [6]. Intervention in IH depends on the size, location, age of the lesion or patient, and potential sequelae of the lesion [7]. Most infantile hemangioma does not require any treatment, as it can regress on its own. However, high-risk lesions, such as those occurring on the face with >5 cm of diameter, located in the central face area, and bulky face lesions, can cause functional compromise, ulceration, and risk of permanent disfigurement [8]. Treatment of infantile hemangioma includes medication, interventional therapy, and, very rarely, surgical therapy.

Using the open rhinoplasty approach for excision in the pediatric group is considered challenging. The key to ideal reconstruction is the preservation of skin and as much healthy soft tissues as possible, as well as avoiding steroid use, topically or systemically along with cryotherapy preoperatively, as it disrupts the integrity of the skin [9]. In addition, a multidisciplinary team approach is recommended especially for patients with recurrent lesions, including dermatologists, radiologists, and vascular and plastic surgeons, to deliver the best management plan. Facial hemangiomas pose a special challenge with high psychological stress for a growing child in which watchful waiting might not be the best option in many cases [10].

## Conclusions

Hemangiomas are very common in the pediatric population with several management options. This study presents a rare case of utilizing the open rhinoplasty technique in removing and reconstructing the facial hemangioma of a 10-year-old patient. This approach suggests a positive aesthetic result in minimizing facial scarring after surgery. Considering the limited report of this approach, further clinical studies especially comparing the long-term effects across different age populations, are strongly recommended in reporting this technique, to describe and validate its efficiency and effectiveness.
